# Self-association of an indole based guanidinium-carboxylate-zwitterion: formation of stable dimers in solution and the solid state

**DOI:** 10.3762/bjoc.6.3

**Published:** 2010-01-14

**Authors:** Carolin Rether, Wilhelm Sicking, Roland Boese, Carsten Schmuck

**Affiliations:** 1 Institute of Organic Chemistry, Faculty of Chemistry, University of Duisburg-Essen, Universitätsstraße 7, 45141 Essen, Germany; 2 Institute of Inorganic Chemistry, Faculty of Chemistry, University of Duisburg-Essen, Universitätsstraße 7, 45141 Essen, Germany

**Keywords:** dimerisation, molecular recognition, self-assembly, supramolecular chemistry, zwitterions

## Abstract

The indole based zwitterion **2** forms stable dimers held together by H-bond assisted ion pairs. Dimerisation was confirmed in the solid state and studied in solution using dilution NMR experiments. Even though zwitterion **2** forms very stable dimers even in DMSO, their stability is lower than of an analogous pyrrole based zwitterion **1**. As revealed by the X-ray crystal structure the two binding sites in **2** cannot be planar due to steric interactions between the guanidinium group and a neighbouring aromatic CH. Hence the guanidinium moiety is twisted out of planarity from the rest of the molecule forcing the two monomers in dimer **2**·**2** to interact in a non-ideal orientation. Furthermore, the acidity of the NHs is lower than in **1** (as determined by UV-pH-titration) also leading to less efficient binding interactions.

## Introduction

The vast majority of supramolecular self-assembling systems known so far form stable assemblies only in non polar solvents such as chloroform, as they mainly rely on hydrogen bonds [1–4]. The design of self-complementary molecules that assemble even in polar solvents is still a challenging task despite all the progress made in this field in recent years. The use of metal-ligand coordination and hydrophobic interactions has proven especially useful in this context [5–11]. We are interested in developing self-complementary zwitterions that from stable aggregates in polar solution based on H-bond assisted ion pair formation. A few years ago we introduced the guanidiniocarbonyl pyrrole carboxylate zwitterion **1** which forms extremely stable dimers not only in the solid state but also in polar solution [12]. In DMSO the stability is too large to evaluate with an estimated association constant of *K*_ass_> 10^10^ M^−1^. Even in water dimerisation still takes place (*K*_ass_ = 170 M^−1^) [13]. The stability of the dimer **1**·**1** is significantly larger than the simple Coulomb-interactions of point charges, suggesting that indeed the formation of directed, H-bond assisted salt-bridges is crucial. Zwitterion **1** combines in a near perfect fit geometrical self-complementarity with the possibility to form two salt-bridges assisted by a network of six H-bonds. The superior stability of **1**·**1** compared to analogous zwitterions based on other aromatic scaffolds such as benzene or furan instead of pyrrole or with an amidinium cation instead of a guanidinium cation was also confirmed by DFT calculations [14]. Zwitterion **1** has thus found widespread application in the formation of self-assembled nanostructures such as vesicles or supramolecular polymers [15–17].

We have now synthesized and studied the indole based zwitterion **2**, a close analogue of **1**. In **2** the guanidinium group is not acylated as in **1** but conjugated to an aromatic ring. Compared to the parent guanidinium cation, in both cases the acidity of the NHs is significantly increased due to the −M effect of the carbonyl group or the aromatic ring, respectively, thus facilitating the formation of H-bond assisted ion pairs [18–19]. Apart from the increased acidity of the NHs in **1** and **2**, also the geometric shape of **2** is very similar to **1** at least based on the inspection of simple models. It was therefore expected that the new zwitterion **2** might form dimers with similar stability to **1**, increasing our repertoire of self-complementary binding motifs that efficiently self-assemble in polar solution. And indeed we could show that zwitterion **2** is able to form highly stable dimers in polar solution and in the solid state as well. However, dimer **2**·**2** is significantly less stable than dimer **1**·**1**. Possible reasons for this decreased stability are discussed.

**Figure 1:**
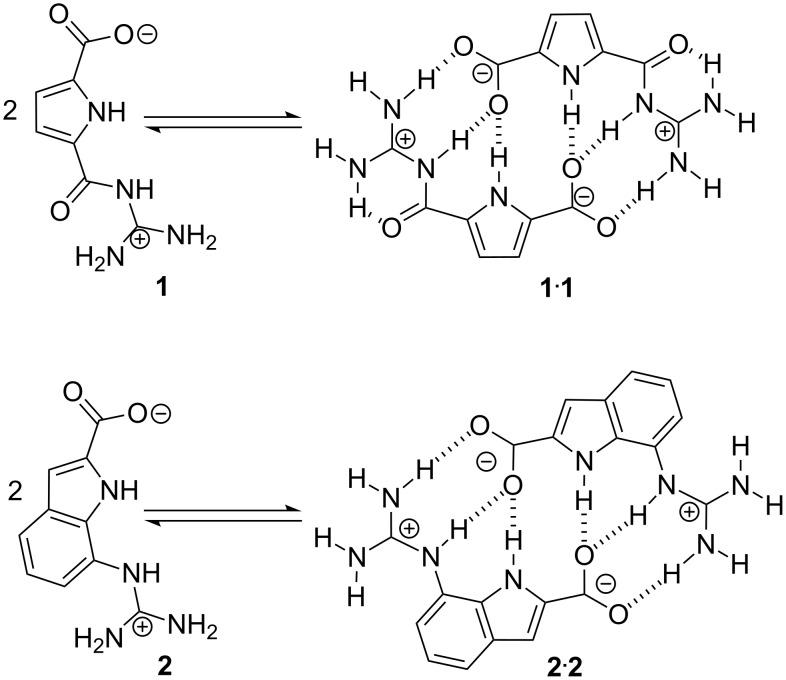
Self-assembly of zwitterion **1** to give dimer **1**·**1** and self-assembly of zwitterion **2** to give dimer **2**·**2** – both using the same intermolecular interactions: a pattern of six H-bonds and two salt bridges.

## Results and Discussion

**Scheme 1:**
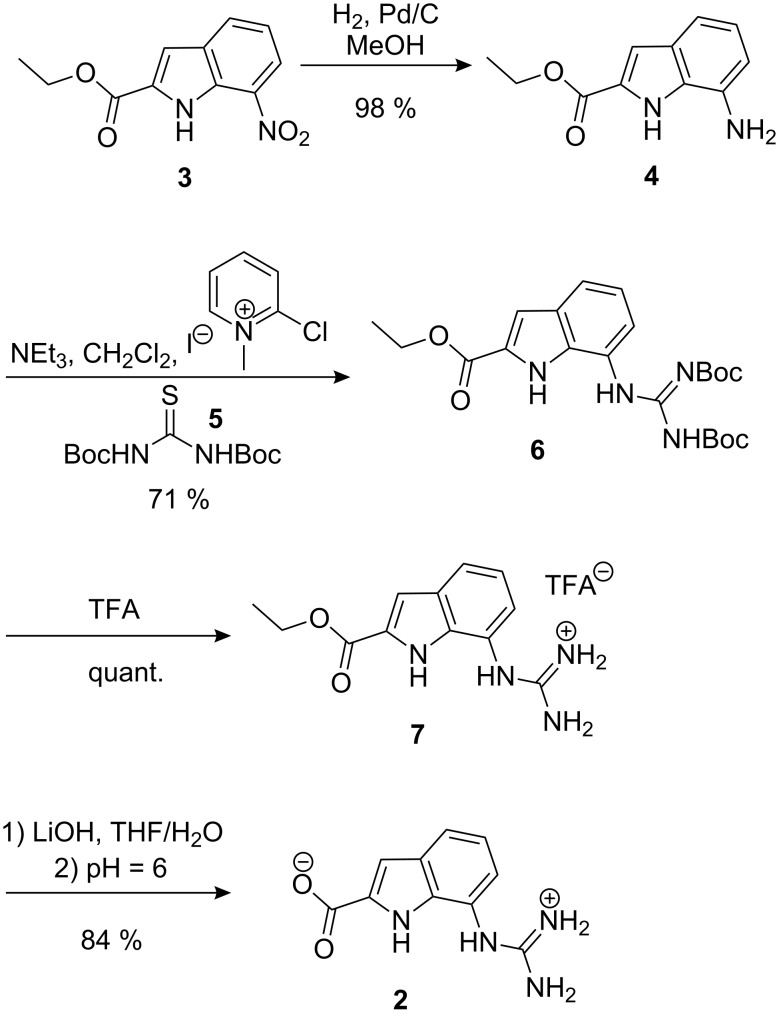
Synthesis of zwitterion **2**.

The indole zwitterion **2** was prepared by a four-step synthesis (Scheme 1). Commercially available 7-nitro-1*H*-indole-2-carboxylate **3** was reduced by reaction with hydrogen in the presence of Pd/C to provide the amine **4** in a yield of 98%. For the next stept, first, thiourea was *N*-Boc-protected at both amino-functions following a literature procedure [20]. Thiourea was deprotonated with sodium hydride and afterwards reacted with di-*tert*-butyl dicarbonate to give the di-Boc-protected thiourea **5** in 79% yield. The di-Boc-protected thiourea **5** was then reacted with the amine **4** in the presence of Mukaiyama’s reagent [21] and triethylamine as a base, which provided **6** in a yield of 71% [22]. Deprotection of the two Boc-groups was achieved by treatment with TFA and the guanidinium salt **7** was obtained quantitatively. In the last reaction step the ethyl ester in **7** was hydrolysed with lithium hydroxide in a THF/water mixture (THF/water = 4/1). Zwitterion **2** was then obtained after adjustment of the pH to 6 with 1M HCl in a yield of 84% as a light brown crystalline solid.

**Scheme 2:**
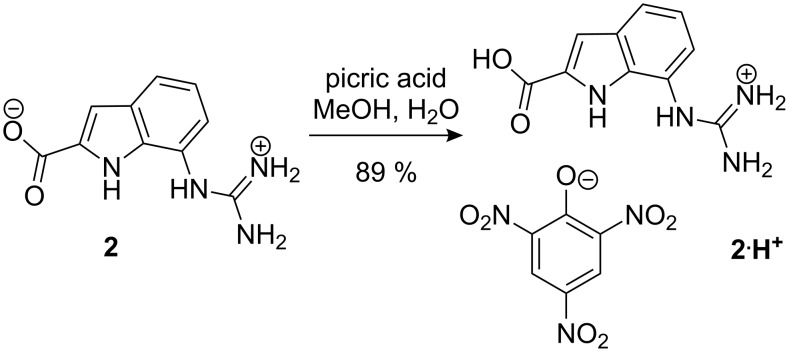
Synthesis of compound **2**·**H^+^**.

For the spectroscopic characterisation and as a reference compound also the picrate salt of **2** was prepared by treating a methanolic solution of **2** with picric acid (Scheme 2). The picrate salt **2**·**H^+^** was isolated in form of a yellow, crystalline solid in 89% yield.

**Figure 2:**
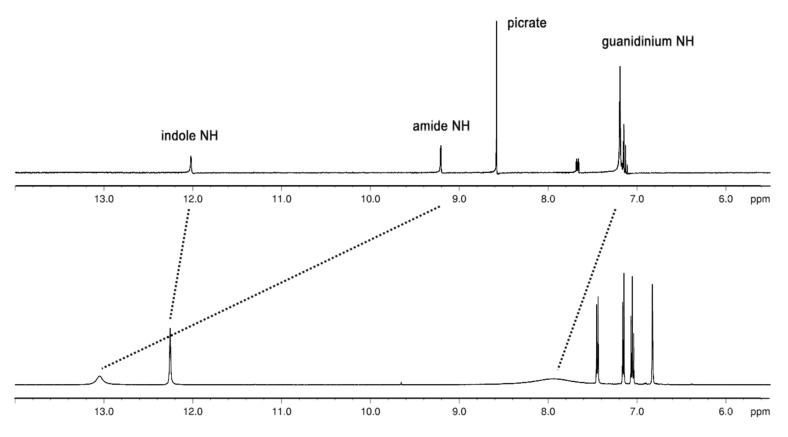
^1^H NMR spectra of zwitterion **2** (bottom) and its protonated form **2**·**H^+^** (top).

While the picrate salt **2**·**H^+^** is moderately soluble in methanol and water, the zwitterionic form of **2** is virtually insoluble in all solvents except DMSO and DMSO-containing solvent mixtures, such as DMSO–MeOH or DMSO–CHCl_3_, so that the dimerisation studies in solution were limited to DMSO. The ^1^H NMR spectrum (Figure 2) of the protonated zwitterion **2**·**H^+^** (picrate salt in [D_6_]DMSO) shows the signals expected for an aromatic guanidinium cation [23]. The four guanidinium NH_2_ protons have a chemical shift of δ = 7.19, whereas the NH of the guanidinium group shows up at δ = 9.21 and the indole NH at δ = 12.06. The signals were assigned based on 2D NMR experiments.

The ^1^H NMR spectrum of zwitterion **2** is significantly different. Especially the NH signals are shifted downfield. The indole NH is shifted downfield by 0.2 ppm and appears at δ = 12.26 and the four guanidinium NH_2_ are shifted to δ = 8.00 ppm. Most significantly the NH of the guanidinium group is shifted downfield by nearly 4 ppm from δ = 9.21 to δ = 13.07 pm. A similar dramatic downfield shift was observed for the guanidinium amide NH of zwitterion **1** upon dimer formation [12–13].

Hence, the downfield shifts in the spectrum of zwitterion **2** relative to the protonated form **2**·**H^+^** are most likely also due to the formation of a H-bonded ion pair which can only take place intermolecularly due to the rigidity of **2**. The similarity of the shift changes with those of zwitterion **1** suggests that dimerisation takes place.

**Figure 3:**
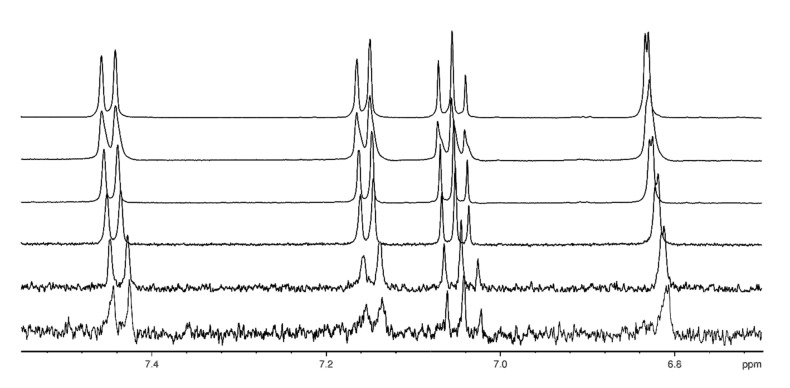
Part of the ^1^H NMR spectrum of **2** in [D_6_]DMSO showing the complexation-induced shifts of the indole CH protons (concentration from bottom to top: 0.4, 1, 6, 12, 25 and 50 mM).

The stability of these dimers was determined by an NMR dilution experiment. To obtain the binding constant for the dimerisation, we studied the concentration dependence of the ^1^H NMR spectrum of **2** in a concentration range from 0.25 to 100 mM in [D_6_]DMSO. The ^1^H NMR shifts are concentration-dependent as expected for a dimerisation (Figure 3).

**Figure 4:**
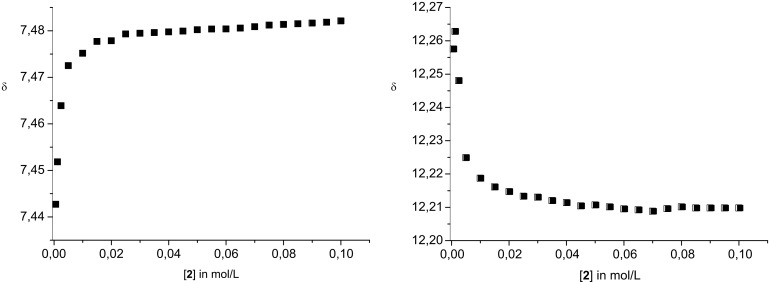
Representative binding isotherm of the aromatic proton d (left) and the indole NH proton (right).

**Figure 5:**
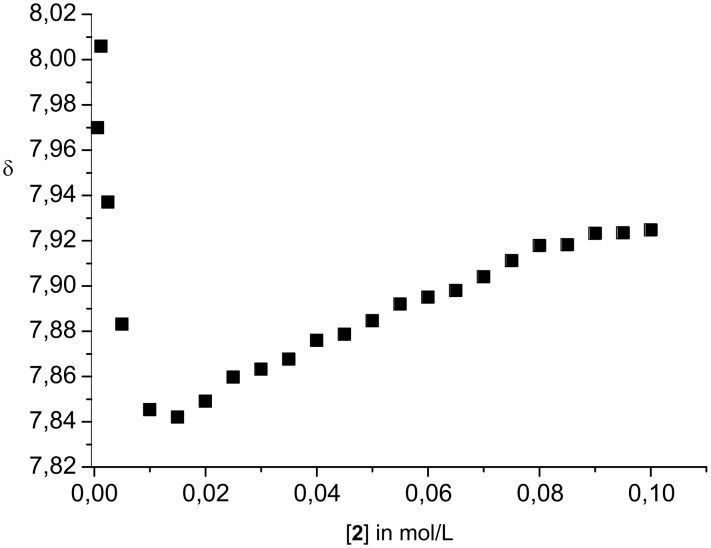
Binding isotherm of the guanidinium NH_2_ protons.

As the binding isotherms show (Figure 4), even at concentrations > 10 mM dimerisation is mostly complete. This suggests very large stability of the dimers even in DMSO. In agreement with this, a quantitative data analysis provided a dimerisation constant *K*_ass_ > 10^5^ M^−1^, too large to be measured accurately by NMR techniques. Similar observations were made earlier for zwitterion **1**. However, for **1** the estimated stability in DMSO was even higher. Interestingly, at higher concentrations the formation of larger aggregates also seems to occur. For example, the signal for the guanidinium NH_2_ protons shows a second shift change at concentrations > 20 mM. First, the signal is shifted to lower field due to the dimerisation, and then a smaller upfield shift is observed (Figure 5). This could be indicative of a second association process in which the dimers **2**·**2** start to interact at concentration > ca. 15 mM. However, the exact nature of these larger aggregates is unclear at the moment.

**Figure 6:**
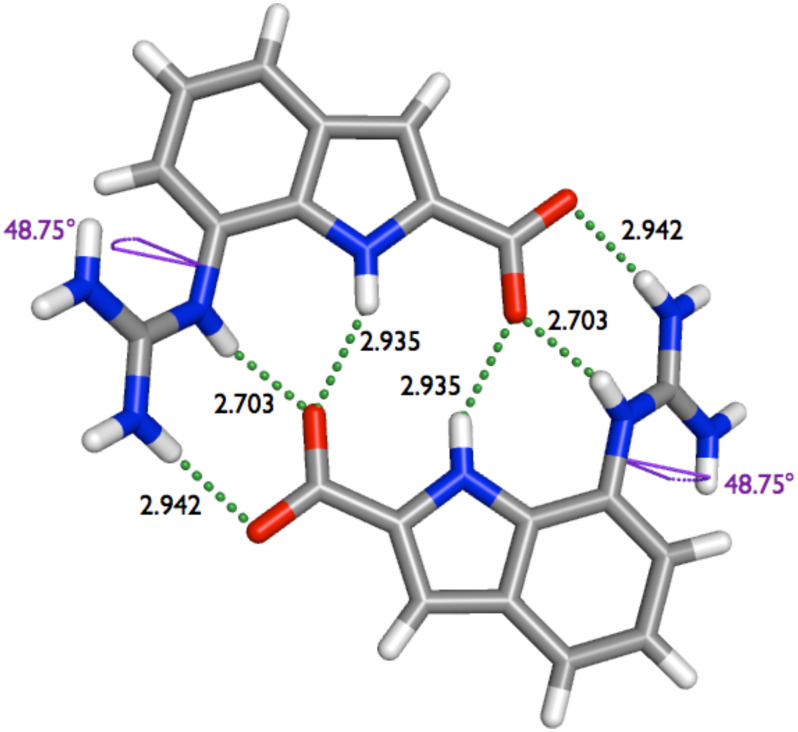
Crystal structure of dimer **2**·**2** with hydrogen bond distances (Å) and dihedral angles.

We were able to determine the solid state structure of **2**. X-ray quality crystals of compound **2** were obtained by slow evaporation of a dimethyl sulfoxide solution. X-ray crystallography confirmed the formation of head-to-tail dimers, which are held together by the formation of two salt bridges assisted by a network of six hydrogen bonds (Figure 6). The hydrogen bond distances between the aromatic N^...^O (2.703 Å), the guanidinium N^...^O (2.942 Å) and the indole N^...^O (2.935 Å) are all rather short.

**Figure 7:**
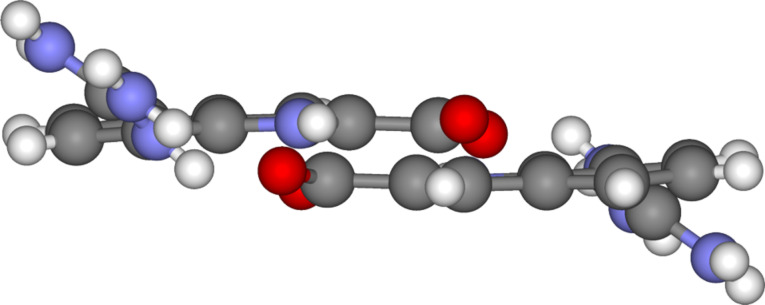
Side view of dimer **2**·**2** in the solid state.

However, the distances are larger than the corresponding distances in dimer **1**·**1**: the amide N^...^O (2.679 Å), the guanidinium N^...^O (2.854 Å), and the pyrrole N^...^O (2.731 Å) distances in dimer **1**·**1** are even shorter than in dimer **2**·**2**. The main difference between **1**·**1** and **2**·**2** is however that the dimers **2**·**2** are not completely planar. Zwitterion **2** itself is not planar, but the guanidinium group is twisted out of planarity by 48.75° (Figure 7). Also the two molecules within the dimer are not within the same plane but slightly offset (by 1.050 pm). This is a consequence of the twisted guanidinium group. To allow optimal interaction of the carboxylate with the NHs of the guanidinium group the second molecule has to be a little bit out of plane of the first, which results in longer hydrogen bond distances for the guanidinium N^...^O and the indole N^...^O (Figure 7) and less favorable H-bond angles within the dimer (164.78° for the outer and 148.97° for the inner guanidinium NH-bonds and 141.37° for the indole NH-bond).

**Figure 8:**
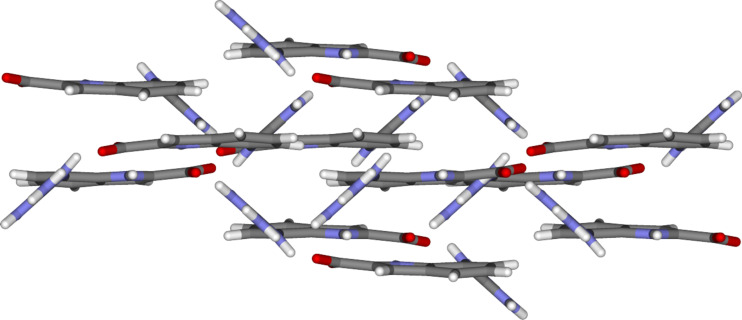
Part of the crystal lattice of zwitterion **2**.

Within the crystal lattice the molecules of **2** are arranged in parallel planes held together most likely by aromatic stacking interactions: The centroid-centroid distance of two indoles is 3.636 Å. Furthermore, the “backside” of the out of plane twisted guanidinium cation also interacts with the carboxylate group one plane below (Figure 8). The corresponding hydrogen bond distances are 2.790 Å and 2.922 Å, respectively, and are therefore similar to the hydrogen bond distances within the dimer.

**Scheme 3:**
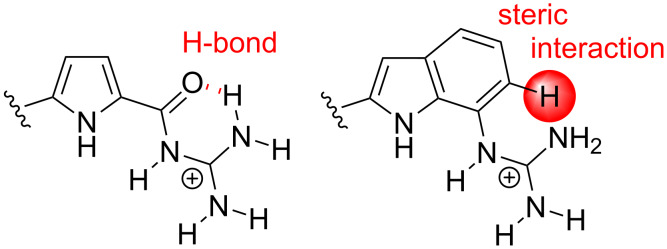
An attractive H-bond in **1** (left) is replaced by a repulsive steric interaction in **2** (right).

The main difference between the pyrrole zwitterion **1** and the indole zwitterion **2** is hence the non-planar, twisted structure of the latter. This is most likely due to steric interactions with the neighboring aromatic C-H bond (Scheme 3). In the pyrrole zwitterion **1** this position is occupied by the carbonyl oxygen which forms an H-bond to the guanidinium moiety and thus actually helps to keep the molecule planar. This amide group in **1** is replaced by the aromatic benzene ring in **2**, thereby replacing an attractive H-bond with a repulsive steric interaction.

**Figure 9:**
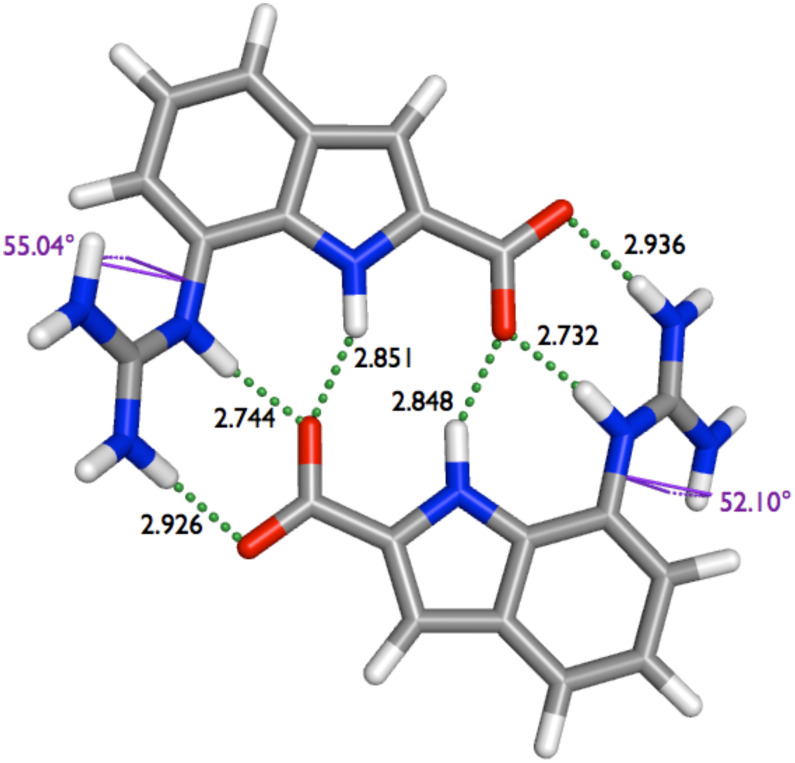
Energy-minimized structure for dimer **2**·**2** with hydrogen bond distances (Å) and dihedral angles.

This twisted, non-planar structure of dimer **2**·**2** is also reproduced by DFT calculations. Geometry optimizations were performed with the Gaussian03 program package using the M05-2X/6-311+G** basis set [24]. In all calculations DMSO as a solvent was included (CPCM, 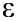 = 48) [25–26]. The optimization revealed the twisted dimer, which fits quite well to the X-ray structure. Though the calculated structure of dimer **2**·**2** is not completely symmetric like the X-ray structure, all the hydrogen bond distances, as well as the torsion angle match pretty well (Figure 9). In the solid state structure, the hydrogen bond distances between the aromatic N^...^O (2.703 Å), the guanidinium N^...^O (2.942 Å) and the indole N^...^O (2.935 Å) are quite short, as mentioned above. The torsion angle between the aromatic scaffold and the guanidinium group is 48.75°. The DFT calculation give an average dihedral angle of 53.57° and lead to the following averaged hydrogen bond distances: 2.738 Å (aromatic N^...^O), 2.931 Å (guanidinium N^...^O) and 2.850 (indole N^...^O).

Hence, the good agreement of the observed structure in the solid state and the calculated structure obtained from DFT calculations suggests that the level of theory used in these calculations describes the dimer with sufficient accuracy. We therefore also calculated the enthalpy values for the dimerisation process of zwitterion **2** and of **1**, respectively, as the experimental values were too large to measure them accurately in DMSO (as mentioned above). The calculated stability of dimer **2**·**2** is significantly lower than for the pyrrole zwitterion **1**: ΔH −54 kJ/mol and −85 kJ/mol, respectively. Hence, even though the bonding interactions in dimer **1**·**1** and **2**·**2** are temptingly similar the latter is only two third as stable as the former.

This difference in stability is most likely due to the non-ideal geometry of the H-bonded ion pairs and reflects the importance of planarity in zwitterion **1** for an effective dimerisation. Due to the twisted guanidinium groups in **2** the two monomers in dimer **2**·**2** are not in-plane, which leads to less efficient interactions. Also as mentioned above, the guanidinium group in zwitterion **2** is directly attached to the aromatic indole scaffold, whereas it is acylated in **1**. Though the overall structure looks similar, this replaces an attractive H-bond which also help to planarize zwitterion **1** by a repulsive steric interaction in **2**, which is responsible for its non-planar structure.

Furthermore, the p*K*_a_ value of the two guanidinium groups as well is an important factor for the stability of the dimers. While simple guanidinium cations as in arginine have a p*K*_a_ of 13.5, the p*K*_a_ of the acylguanidinium group in **1** was measured by UV-pH-titration to be 6.3 ± 0.1. Analysis of the pH dependent UV spectral changes was performed using the Specfit/32 software program from Spectrum Software Associates. However, the p*K*_a_ of the guanidinium group in **2** also obtained from a UV-pH-titration is significantly larger with p*K*_a_ = 10.6 ± 0.1. Hence, the lower acidity of the NHs in **2** is a second important factor leading to the overall reduced stability of dimer **2**·**2**.

## Conclusion

In conclusion, we have presented the synthesis of a new indole based zwitterion **2**, a close analogue of the 5-(guanidiniocarbonyl)-1*H*-pyrrole-2-carboxylate (**1**) which we recently introduced as one of the most stable self-complementary simple molecules known so far. Both dimers rely on the same intermolecular interactions, two salt-bridges assisted by a very similar network of six H-bonds. We could show here that zwitterion **2** also self-assembles into stable dimers in the solid state and also solution (*K*_ass_ > 10^5^ M^−1^ in DMSO). However, DFT calculations suggest that the dimers are significantly less stable than dimer **1**·**1** despite the overall similarity of the binding interactions. The calculated dimerisation enthalpy for dimer **2**·**2** is only 66% of that for dimer **1**·**1**. This is most likely due to two reasons. As the solid state structure shows, the two binding sites in **2**·**2** are not coplanar, but the guanidinium moiety is twisted out of plane of the aromatic ring. This forces the two zwitterions in the dimer also to be out of plane leading to less efficient interactions between them. Furthermore, the NHs in **2** are significantly less acidic than in **1** which also reduces the stability of H-bonded ion pairs. Hence, geometric as well as electronic fit is the important factor controlling the stability of aggregates obtained from such self-complementary molecules. Nevertheless, zwitterion **2** is an efficient self-assembling molecule. This indole guanidinium cation might also be an interesting binding motif for the recognition of oxoanions by indole based receptors [27–29], similar to our guanidiniocarbonyl pyrrole cation [30–32].

## Experimental

**General Remarks:** Solvents were dried and distilled before use. The starting materials and reagents were used as obtained from Aldrich or Fluka. ^1^H and ^13^C NMR spectra were recorded with a Bruker Avance 400 spectrometer. The chemical shifts are reported relative to the deuterated solvents. The ESI-mass spectra were recorded with a Finnigan MAT 900 S spectrometer. IR spectra were recorded by measuring the Attenuated Total Reflectance (ATR). Melting points are not corrected. The pH values were measured with a Knick pH meter 766 Calimatic at 25 °C. UV spectra were measured in 10 mm rectangular cells with a Jasco V660 spectrometer.

**Ethyl 7-amino-1*H*-indole-2-carboxylate (4):** A mixture of ethyl 7-nitro-1*H*-indole-2-carboxylate (**3**; 200 mg, 0.85 mmol) and Pd/C (20 mg) in methanol (40 mL) was hydrogenated at ambient temperature for 1.5 h. The mixture was filtered over Celite to remove Pd/C, and the solvent was evaporated to give the desired product **4** (170 mg, 0.83 mmol, 98%) as a colourless solid: mp 146 °C; ^1^H NMR (400 MHz, [D_6_]DMSO, 25 °C): δ = 1.34 (t, 3H), 4.34 (q, 2H), 5.38 (s, 2H), 6.41 (dd, 1H), 6.78-6.86 (m, 2H), 7.02 (d, 1H), 11.40 (bs, 1H) ppm; ^13^C NMR (100 MHz, [D_6_]DMSO, 25 °C): δ = 14.3, 60.3, 106.5, 108.1, 109.5, 121.5, 126.2, 127.3, 127.6, 134.6, 161.5; IR (KBr): ν = 3329 (s), 2996 (w), 2939 (w), 1668 (s), 1250 (s), 1215(s) cm^−1^; HR-MS (ESI) calcd for [M+H]^+^: 205.0972; found 205.0979.

***N*,*N*’-Di-(*tert*-butoxycarbonyl)thiourea (5):** To a stirred solution of thiourea (570 mg, 7.50 mmol) in dry tetrahydrofuran (150 mL) sodium hydride (1.35 g, 33.80 mmol, 60% in mineral oil) was added under argon atmosphere at 0 °C (ice bath). After 5 min the ice bath was removed and the mixture was stirred for additional 10 min at ambient temperature. The mixture was cooled to 0 °C again and di-*tert*-butyl dicarbonate (3.60 g, 16.50 mmol) was added. After 40 min of stirring at 0 °C the ice bath was removed and the mixture was stirred for additional 3 h at ambient temperature. The reaction was quenched by adding an aqueous saturated solution of NaHCO_3_ (10 mL). Water (200 mL) was added and the reaction mixture was extracted with ethyl acetate (3 × 75 mL). The collected organic layers were dried over MgSO_4_, filtered and evaporated to dryness. The white solid was purified by flash column chromatography on silica gel (hexane/ethyl acetate = 1 : 1 + 0.5% triethylamine) to give *N*,*N*’-di-(*tert*-butoxycarbonyl)thiourea (**5**, 1.63 g, 5.92 mmol, 79%) as a colourless solid: mp 130 °C; ^1^H NMR (400 MHz, [D_6_]DMSO, 25 °C): δ = 1.44 (s, 9H), 1.45 (s, 9H), 8.96 (s, 1H), 9.14 (s, 1H) ppm; ^13^C NMR (100 MHz, [D_6_]DMSO, 25 °C): δ = 27.6, 82.5, 150.5, 178.7; IR (KBr): ν = 3160 (s), 2987 (m), 2933 (m), 1767 (m), 1718 (m), 1128 (s) cm^−1^; HR-MS (ESI) calcd for [M+H]^+^: 277.1217; found 277.1056.

**Ethyl 7-{*N*,*N*’-bis-[*tert*-(butoxycarbonyl)guanidino]}-1*H*-indole-2-carboxylate (6):** To a solution of ethyl 7-amino-1*H*-indole-2-carboxylate (**4**, 130 mg, 0.65 mmol), *N*,*N*’-di-(*tert*-butoxycarbonyl)thiourea (**5**, 185 mg, 0.65 mmol) and triethylamine (0.35 mL, 2.44 mmol) in dry dichloromethane (30 mL) was added 2-chloro-1-methyl-pyridinium iodide (297 mg, 1.14 mmol) at 0° C and the mixture was stirred for 30 min. The ice bath was removed and the reaction mixture was stirred at ambient temperature for 24 h. The solvent was removed under reduced pressure, and the residue was purified by flash column chromatography on silica gel (ethyl acetate/hexane = 2:3) to give **6** (206 mg, 0.46 mmol, 71%) as a colourless solid: mp 144 °C; ^1^H NMR (400 MHz, [D_6_]DMSO, 25 °C): δ = 1.28 (s, 9H), 1.34 (t, 3H), 1.54 (s, 9H), 4.36 (q, 2H), 7.09 (t, 1H), 7.20 (d, 2H), 7.60 (d, 1H), 9.73 (s, 1H), 11.56 (bs, 1H), 11.95 (bs, 1H) ppm; ^13^C NMR (100 MHz, [D_6_]DMSO, 25 °C): δ = 14.3, 27.8, 27.8, 60.5, 78.4, 82.9, 108.4, 120.2, 120.9, 121.8, 122.8, 127.8, 128.3, 133.5, 152.0, 155.0, 161.2, 162.8; IR (KBr): ν = 3136 (w), 2974 (w), 2930 (w), 1717 (m), 1636 (m), 1360 (m) cm^−1^; HR-MS (ESI) calcd for [M+Na]^+^: 469.2058; found 469.2096.

**2-(Ethoxycarbonyl)-1*H*-indole-7-guanidinium trifluoroacetate (7):** Trifluoroacetic acid (3 mL) was added to the ethyl 7-[*N*,*N*’-bis-(*tert*-butoxycarbonyl)guanidino]-1*H*-indole-2-carboxylate (**6**, 170 mg, 0.39 mmol), and the reaction mixture was stirred at room temperature for 2 h. The excess trifluoroacetic acid was removed in vacuo to give **7** as a colourless solid (140 mg, 0.39 mmol, 100%): mp > 240 °C; ^1^H NMR (400 MHz, [D_6_]DMSO, 25 °C): δ = 1.35 (t, 3H), 4.36 (q, 2H), 7.13-7.26 (m, 7H), 7.69 (d, 1H), 9.29 (s, 1H), 12.11 (s, 1H) ppm; ^13^C NMR (100 MHz, [D_6_]DMSO, 25 °C): δ = 14.3, 60.6, 108.7, 112.0, 120.7, 121.8, 122.8, 128.3, 128.9, 133.5, 156.4, 161.1; IR (KBr): ν = 3298 (w), 3193 (w), 3101 (w), 2955 (w), 1699 (m), 1671 (s), 1255 (s) cm^−1^; HR-MS (ESI) calcd for [M+H]^+^: 247.1190; found 247.1215.

**7-Guanidinio-1*H*-indole-2-carboxylate (2):** To a solution of the trifluoroacetate salt **7** (130 mg, 0.53 mmol) in water/THF (1/4; 15 mL) LiOH·H_2_O (223 mg, 5.30 mmol) was added. The reaction mixture was heated to 40 °C and stirred for 8 h. The solvent was removed under reduced pressure and the residue was dissolved in water (20 mL). The solution was acidified dropwise with hydrochloric acid (0.1 molar) until a yellow solid precipitated at a pH = 6. The solid was filtered and to remove inorganic salts again suspended in water (25 mL), some dioxane (5 mL) was added. The mixture was heated to reflux for 40 min. The residue was filtered, and washed with water and afterwards with diethyl ether. The residue was dried in vacuo to give **2** as a light brown solid (98 mg, 0.44 mmol, 84%): mp > 240 °C; ^1^H NMR (400 MHz, [D_6_]DMSO, 25 °C): δ = 6.82 (s, 1H), 7.05 (t, 1H), 7.15 (d, 1H), 7.43 (d, 1H), 7.99 (bs, 4H), 12.26 (bs, 1H), 13.07 (bs, 1H) ppm; ^13^C NMR (100 MHz, [D_6_]DMSO, 25 °C): δ = 103.4, 113.0, 118.1, 119.7, 122.0, 128.1, 129.6, 135.9, 155.9, 166.4; IR (KBr): ν = 3327 (m), 3086 (m), 3724 (m), 1397 (s), 737 (s) cm^−1^; HR-MS (ESI) calcd for [M+H]^+^: 219.0877; found 219.0884.

**(2-Carboxy-1*H*-indole-7-yl)guanidinium picrate (2·H^+^):** To a suspension of the zwitterion **2** (20 mg, 0.09 mmol) in methanol (4 mL) a saturated solution of picric acid in water (6 mL) was added and the mixture was stirred for 24 h at ambient temperature. The picrate salt crystallized and was filtered, washed several times with methanol, and dried to provide the yellow solid **2**·**H^+^** (35 mg, 0.08 mmol, 89%): mp > 240 °C; ^1^H NMR (400 MHz, [D_6_]DMSO, 25 °C): δ = 7.11-7.20 (m, 8H), 7.67 (d, 1H), 8.58 (s, 2H), 9.21 (s, 1H), 12.02 (s, 1H), 13.23 (bs, 1H) ppm; ^13^C NMR (100 MHz, [D_6_]DMSO, 25 °C): δ = 120.5, 124.1, 125.2, 129.1, 129.2, 141.9, 156.3, 160.9; IR (KBr): ν = 3200(w), 1674 (w), 1554 (m), 1336 (m) cm^−1^; HR-MS (ESI) calcd for [M+H]^+^: 219.0877; found 219.0884.

### X-ray Crystallographic Data

**Crystal structure of 2:** C_10_H_10_N_4_O_2_, colourless crystals, dimensions 0.16 × 0.13 × 0.10 mm^3^, measured with a Bruker D8 KAPPA series II with APEX II area detector system at 100 K; *a* = 12.1695 (5) Å, *b* = 7.1061 (3) Å, *c* = 12.3061 (4) Å, *V* = 985.45 (7) Å^3^, *Z* = 4, *ρ* = 1.471 g/cm^3^, space group *P*2_1_/*n*, 7030 intensities measured (θ_max_ = 28.33°), 2458 independent (*R*_(int)_ = 0.0279), 2061 observed, structure solution by direct methods and refinement of 145 parameters on *F^2^* with the Bruker software package SHELXTL Vers. 2008/4/(c) 2008, *R*_1_ = 0.0485, *ωR*_2_ (all data) = 0.1111, Gof = 1.053, max electron density 0.407 e Å^−3^.
